# Visualization of Protein Folding Funnels in Lattice Models

**DOI:** 10.1371/journal.pone.0100861

**Published:** 2014-07-10

**Authors:** Antonio B. Oliveira, Francisco M. Fatore, Fernando V. Paulovich, Osvaldo N. Oliveira, Vitor B. P. Leite

**Affiliations:** 1 Departamento de Física, Instituto de Biociências, Letras e Ciências Exatas, Universidade Estadual Paulista, Sao José do Rio Preto, São Paulo, Brazil; 2 Instituto de Ciências Matemáticas e Computação, Universidade de São Paulo, São Carlos, São Paulo, Brazil; 3 Instituto de Física de São Carlos, Universidade de São Paulo, São Carlos, São Paulo, Brazil; Weizmann Institute of Science, Israel

## Abstract

Protein folding occurs in a very high dimensional phase space with an exponentially large number of states, and according to the energy landscape theory it exhibits a topology resembling a funnel. In this statistical approach, the folding mechanism is unveiled by describing the local minima in an effective one-dimensional representation. Other approaches based on potential energy landscapes address the hierarchical structure of local energy minima through disconnectivity graphs. In this paper, we introduce a metric to describe the distance between any two conformations, which also allows us to go beyond the one-dimensional representation and visualize the folding funnel in 2D and 3D. In this way it is possible to assess the folding process in detail, e.g., by identifying the connectivity between conformations and establishing the paths to reach the native state, in addition to regions where trapping may occur. Unlike the disconnectivity maps method, which is based on the kinetic connections between states, our methodology is based on structural similarities inferred from the new metric. The method was developed in a 27-mer protein lattice model, folded into a 3×3×3 cube. Five sequences were studied and distinct funnels were generated in an analysis restricted to conformations from the transition-state to the native configuration. Consistent with the expected results from the energy landscape theory, folding routes can be visualized to probe different regions of the phase space, as well as determine the difficulty in folding of the distinct sequences. Changes in the landscape due to mutations were visualized, with the comparison between wild and mutated local minima in a single map, which serves to identify different trapping regions. The extension of this approach to more realistic models and its use in combination with other approaches are discussed.

## Introduction

Understanding the processes leading to a protein folding into its native (functional) state is one of the important problems in molecular biophysics. In the 1960s, Anfinsen hypothesized that a protein in its native state and under physiological conditions would adopt such a structure with the lowest possible energy [1]. Though this hypothesis turned out to be correct, no explanation was offered to explain the large range of characteristic folding times, which may vary from milliseconds to seconds. In what became known as the Levinthal Paradox, in 1969 Levinthal argued that, due to an exponentially large number of states, a random search for the native structure would take cosmological times [2]. The solution to this paradox came from the energy landscape theory [3]–[7], which embeds the statistical nature of the folding process. The folding happens in a very high dimensional space, but in one of the possible descriptions, the complex landscape theory is projected along the reaction folding coordinate. The effective folding landscape topology is like a funnel, which has an energy gradient toward the native state region. This theory explained quantitatively the data for the folding of several proteins [8]–[14], and the funnel topology is correlated with the thermodynamics and kinetics of folding [15]. Many aspects of the folding funnel can be inferred from this approach, such as analysis of conformational maps [16], [17], folding mechanisms involving mutants [18], and topological features in the transition state [19].

In other approaches, local minima are individually addressed and go beyond one-dimensional representation [20], [21]. Visualization of distances between local minima is a very appealing way of showing the underlying structure of the funnel. However, visualizing the local minima poses a significant challenge owing to the multidimensional nature of the system. Among the motivations to investigate the funnel details and its visualization is the potential help in understanding the role of metastable states, kinetic routes and conformational changes associated with protein function [22]–[24]. The visualization of potential and free energy surfaces is not essential for calculating any dynamic or thermodynamic properties, but it can certainly help in providing insights as to what those properties might be [20], [25], [26]. Methods such as Principal Component Analysis (PCA) have been used in funnel visualization for isobutyryl-(ala)_3_-NH-methyl (IAN) [27], where disconnectivity graphs were used to visualize the overall organization of the landscape [28]. The potential energy surface is represented in terms of local minima and the transition states that connect them, providing a convenient coarse-grained representation of the corresponding landscape [29]. This method has been applied to a wide number of systems. For example, Lennard-Jones clusters present multi-funnel characteristics [30]–[32]. Disconnectivity graphs are able to reveal the effects of gatekeepers in the potential energy surface by raising the energies of low-lying minima relative to the global minimum [33]. The diferences in folding efficiencies can also be inferred in proteins with and without frustration for structure based models [34]. Disconnectivity graphs can also be extended for the visualization of free energy landscape, maintaining the description of barriers faithfully [26], [35], [36]. When rate constants are associated with the rearrangements mediated by each transition state, a kinetic transition network can be defined [37], [38]. So the kinetics and thermodynamics of complex transitions can be modeled in terms of transitions between the relevant conformational substates [39]–[41], in which kinetic transition networks are constructed from geometry optimization and molecular dynamics simulations. These examples show that this method overcomes the fundamental limitations of reaction-coordinate-based methods. Most of these approaches emphasize the kinetic path between probed states, and are able to indicate, for example, the funnel aspect of the landscape against a hub-like hypothesis [41].

In this paper we focus on the structural organization of conformations, looking at the difference of contacts in each conformation. We propose a suitable conformation metric that reflects the underlying landscape in which the kinetics takes place. The method is tested in a 27-mer protein lattice model, folded into a 3×3×3 cube, which has been extensively used in protein folding studies [3], [42], [43], and in particular for visualization methods [44]. We restricted the visualization to local minima of regions from around the transition-state to the native state. These partially folded states are the relevant ones in the study of metastable states and function-related conformation changes. The data obtained from computational simulations in a lattice model were projected on a 2D or 3D plot with the Force-Scheme method [45], which allowed us to map the connectivity of conformations (local minima). The choice of a metric is essential in order to reach a sensible connection between the original data and the projection, and it must efficiently distinguish between pairs of conformations. From the analyses, we noted that distinct sequences lead to different patterns, from which folding routes could be established and the effects from mutations could be probed.

## Results and Discussion

The simulation of the folding dynamics probes the conformations associated with local minima within given time intervals. We are interested in mapping the partially folded states, associated with conformations from the transition-state to the native configuration. The transition state was inferred from the free energy as a function of degree of nativeness (see Supporting Information) for the protein-like sequences A, Af, B, C and D. Conformational states are characterized by the energy and non-bonding contact points for each monomer of the sequence. The dataset thus generated is multidimensional, and its visualization requires dimension reduction projection methods. A crucial point for the projection is to establish a metric for the distance between two conformations. We tried several possibilities, including the Minkowski family of metrics [46], of which the Euclidean distance is one example. These did not lead to physically plausible results since the computation of such metrics considers that lack-of-contact comparisons define similar elements. In the lattice case, the absence of contact (“0” comparisons) occurs when two conformations do not present contacts. In this scenario a binary distance is a better choice, *i.e.*, only contacts (“1” comparisons) are relevant.

The measure between two conformations *i* and *j* has to satisfy commutativity and null distance to itself, *i.e.*,
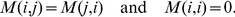
(1)


The structural measure or distance shown to be most effective was the ratio between the dissimilarity 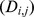 and similarity 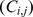 between *i* and *j*, which is equivalent to the ratio between the Jaccard index and the Jaccard distance [47], defined as
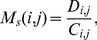
(2)

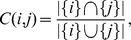


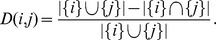




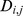
 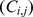 is given by the number of different (common) non-bonded contacts between conformations given by the set of contacts 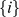 and 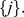 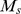 takes into account all the contacts whether they are native or not. Comparing 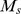 with other variables often used, the usual reaction coordinate 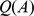 (given by the fraction of native contacts formed in conformation 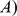 cannot satisfy Eq.(1), since 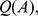 given a native reference 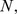 is different from 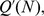 given a reference conformation 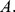 Root Mean Square deviation (RMSD) satisfies the Eq.(1) conditions, but compares the overall conformation, which may not properly account for local details.

One could argue that this topological distance, which could capture static features of the conformation space, may not cope with details of folding. Folding process is an intrinsically dynamic process, which is also the basis of the the discontinuity graphs discussed in the Introduction. Moreover, two structurally similar conformations could differ in terms of the dynamics for folding. We therefore incorporated in the simulations a dynamic measurement defined by
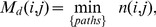
(3)where 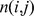 is the number of local minimum intermediates required to go from 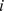 to 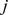 conformations. 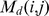 corresponds to the minimum calculated over all the paths going from 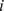 to 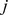 (or vice-versa). The measurement is normalized upon dividing by the largest distance encountered. This approach resembles the method using to determine kinetic transition networks [48]–[50]. In subsidiary simulations we noted that using an effective distance 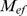 (in Eq.(4)), which takes into account the dynamic measurement, yields essentially the same results as with our initial measurement defined in Eq.2. Therefore the use of the latter appears to embed the underlying landscape of the system.

### Visualizing the folding funnel

**Figure 1:**
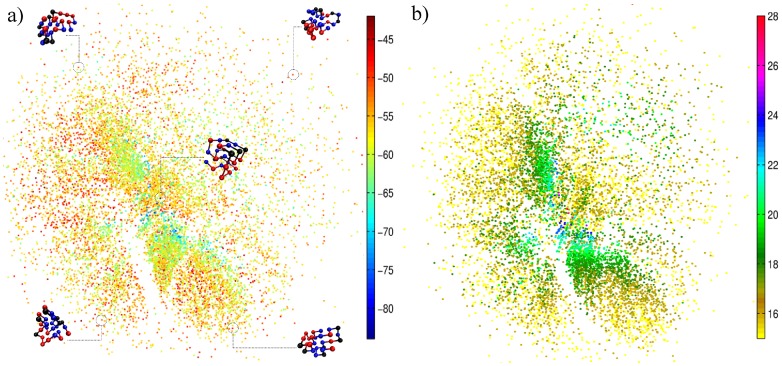
Visualization in 2D of the conformation space for sequence A. Each point represents one conformation (local minimum) and the distance between points refers to the projection of their effective distance. The axis directions do not have any special meaning and have been removed. In (a) the color is associated with the conformation energy. In (b) the color is associated with the reaction coordinate 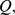 where 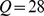 represents the native state.

The protein funnel was obtained by projecting the multidimensional local minima, distributed according to the effective metric distance, onto a 2D surface. The 5 sequences investigated, viz. A, Af, B, C and D, are described in detail in the Methods. Figure 1 shows the funnel representation of sequence A, in which the minima are colored according to conformation energy in Figure 1a, or according to the reaction coordinate 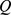 in Figure 1b. The steep convergence to the native state either in energy or 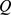 representation is an indicative of the principle of minimal frustration associated with this sequence. The important information is the relative distance between two given points, and the axes were removed because the directions do not have any special meaning. Different regions in the 2D representation can be associated with different partially folded motifs, as shown in Figure 1a. As expected, different time intervals sample different minima, thus yielding varying local minima resolution, but the overall funnel pattern was maintained (see Figures S3, S4, S5 and S6 in the Supporting Information). The pattern preservation for distinct time intervals (in MCs) ensures that the sequence possesses a unique “signature”, with clusters of conformations becoming denser as the number of time intervals decreases (probing more fluctuations). For a 30 MCs interval, in particular, a more refined energy distribution can be visualized with the identification of higher energy conformations when compared with local minima in simulations with larger time intervals.

**Figure 2:**
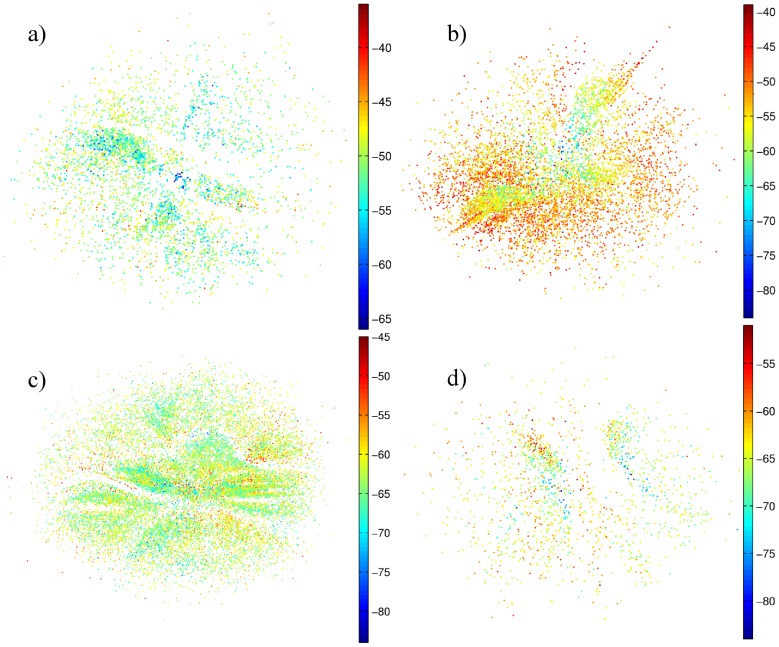
2D visualization for the sequences (a) Af; (b) B; (c) C and (d) D, obtained with a time interval of 1000 MCs.

Figure 2 shows that the funnel landscape obviously depends on the protein sequence, with a unique native structure being represented by a unique funnel landscape. The sequence D, in particular, has a doubly degenerate native state, where the two lowest-energy conformations differ from each other by 5 native contacts. The existence of these two native states is reflected in two clusters of points in Figure 2d. For this sequence, a change from one region (native state) to the other native state requires unfolding (i.e. the need to move towards the periphery in the projection). Note that, for sequences that are difficult to fold (Figure 2a and 2c), the number of conformations with intermediate energy (in the green light blue region) increases considerably, in comparison with the easily-foldable sequences (A and B) (Figure 1 and 2b). By the same token, the sequences with non-efficient folding funnels take a much longer average time to fold, as shown in Figure S2 in the Supporting Information.

**Figure 3:**
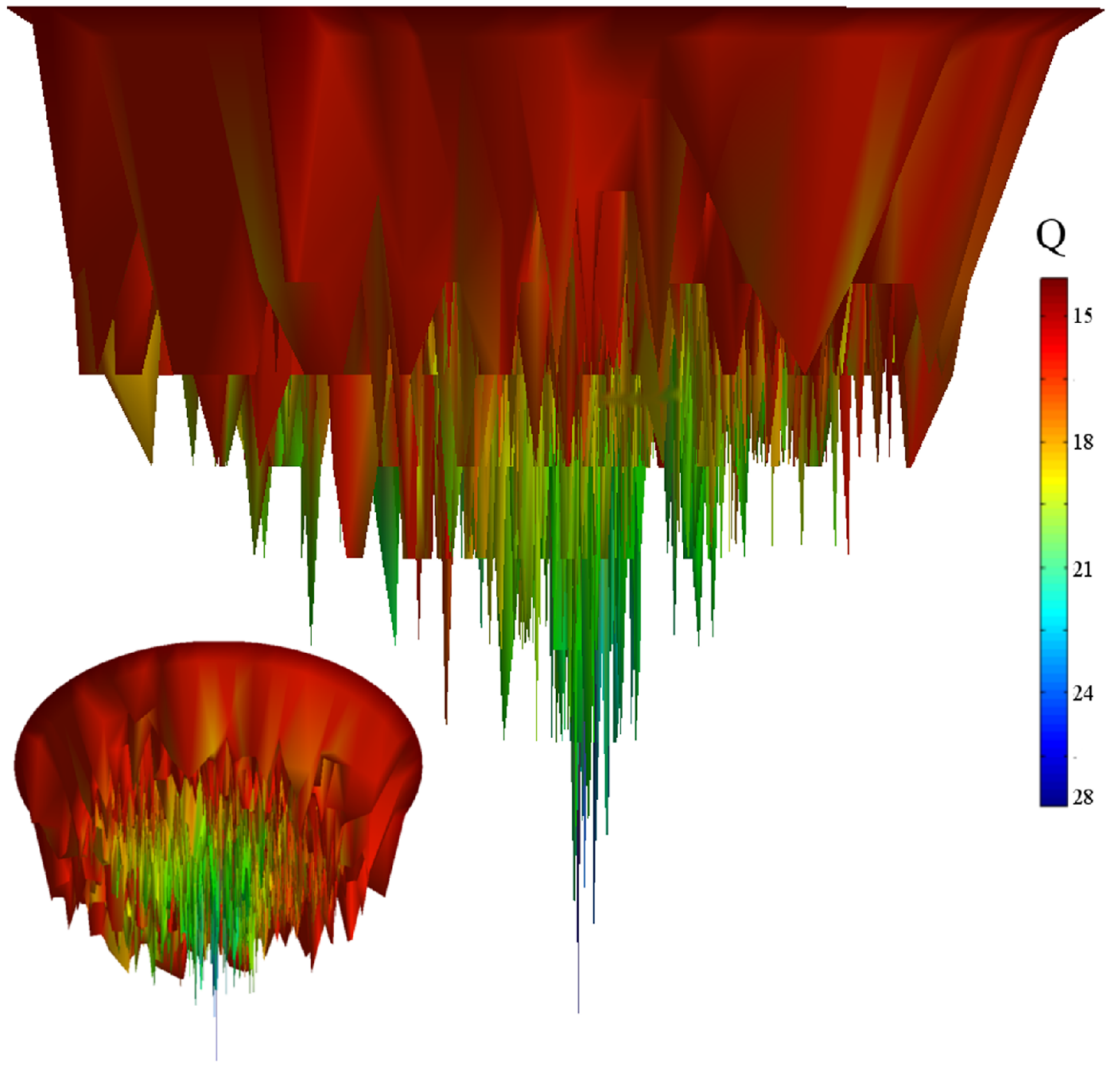
3D visualization of the funnel for sequence A, with two different views. The third axis (depth) of the funnel is associated with the energy of the local minima, and the color map is the reaction coordinate 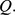

In order to generate a 3D visualization for the funnel, the 2D representation was taken for the 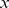 and 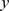 axes, while the energy was taken as the z axis, with the lowest energy value corresponding to the native state. Color encodes the reaction coordinate 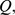 which is the degree of nativeness. Figure 3 shows the 3D picture of the funnel for the sequence A, while the figures for the other sequences are given in Figures S7 and S8 in the Supporting Information. It must be stressed that the result of the projection method is independent of the initial condition of the states in the 2D representation. The native conformation converges to the center of the funnel without any constraint or external force. The global minimum of the system, or native configuration, in the center of the 2D representation reinforces the funnel-like structure of the landscape.

### Folding routes

**Figure 4:**
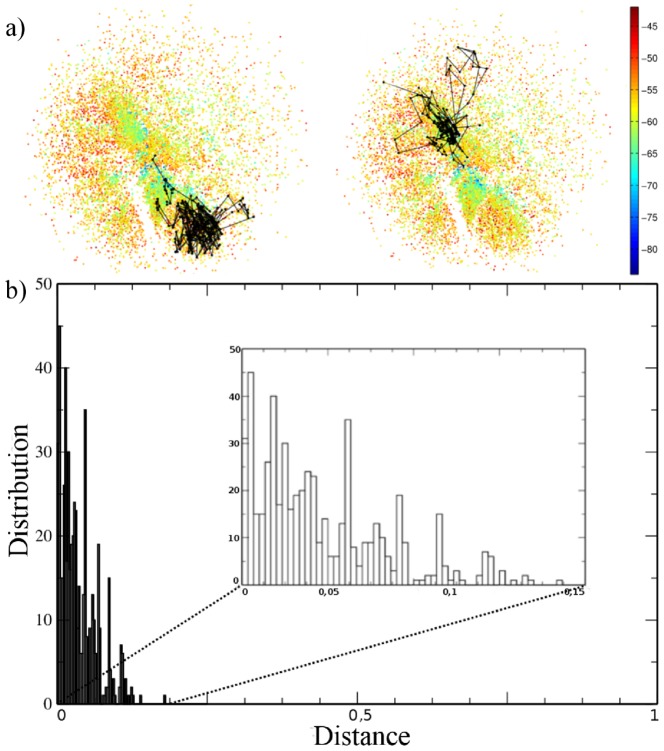
Analysis of folding routes. In (a) Folding routes for the sequence A, where the starting point was a random conformation and the final point corresponds to the native state. In (b) Histogram of the distribution of distances between two subsequent local minima in the 2D representation for very long trajectories.

The 2D and 3D visualizations of the folding funnels appear to confirm that the strategy proposed here is suitable for describing the folding process, but they do not suffice to ensure that the choice of the distance metrics is robust. The latter can be probed by analyzing the folding routes, for in a good funnel representation the folding route has to be represented by a sequence of small steps in the effective funnel representation. Figure 4a shows two routes generated from first passage time simulations, which show mostly small steps between successive minima. The details of this representation can be seen in different folding routes, which probe very distinct regions of the phase space (associated with different partially folded motifs). Also worth mentioning is that the routes do not directly cross the empty regions, but go around them through neighboring connected states. Figure 4b shows that, for sequence A, the distances between two subsequent local minima in the 2D representation are almost always very small, which means that no drastic changes occur in conformation from one minimum to the next. This confirms the robustness of the approach presented here.

### Analysis of a mutation

**Figure 5:**
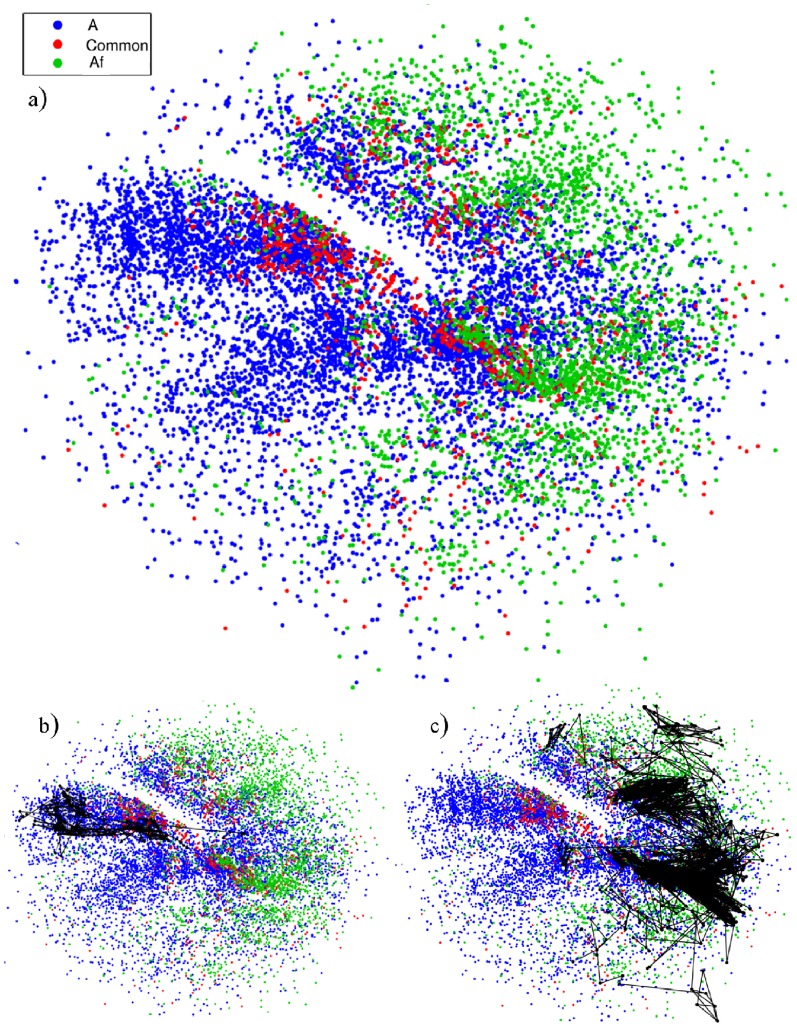
2D Projection of sequence A (blue points) and its mutated form Af (green points), while the points in red are common to both A and Af sequences. An example of a route for each of the sequences is presented: (b) sequence A and (c) sequence Af.

The 2D projection was also used to explore a mutation in sequence A, where two monomers were exchanged to yield a less stable sequence (see Table 1 in the Methods). The effects from the mutation can be evaluated by mapping the data of the two sequences in the same projection. Due to mutation a set of conformations is no longer energetically favorable for the folding. This can be seen in Figure 5a where the whole region on the left is missing for the mutated sequence (green points). One thousand (1000) folding routes were calculated for each sequence, with examples shown in Figures 5b and 5c. In contrast to the wild sequence (A), for the mutated sequence (Af) the routes normally probe a significant part of conformational space before reaching the native state, with 95% of the pathways occurring on the right-hand part of the projection. The mutation stabilizes a different set of local minima, which hinders the folding process and causes a considerable increase in the average folding time (as seen in Figure S2). Note that most of the minima in the mutated sequence do not coincide with those of the wild sequence, thus indicating that they are structurally different, even though they have the same native state.

**Table 1:** Summary of sequences utilised.

Sequences	Zscore†	Representation	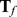
A^‡^	6.75	ABABBBCBACBABABACACBACAACAB	1.89
Af^§^	5.91	ABABBBCBACBABA**C**ACACBA**B**AACAB	1.23
B	8.58	ABCBDBEABBAEBDBCBAABCBDBEAB	1.90
C	5.90	AAAAAABCAACBAABCAAAACCAAAAC	1.95
D	6.27	AAABAAAACABAAABABACABAACABA	1.73

† Zscore is calculated according to methodology described by Dima et al. [58]. ^‡^Sequence design by Shakhnovich et al. [59] which has been used in other studies [42], [43]. ^§^This sequence was obtained through a permutation of two monomers in A, which results in three frustrated contacts in the native structure.

## Conclusions

Visualization was based on the assumption that the distance between two conformations was the ratio between the Jaccard index and the Jaccard distance taking into account all non-bonded contact points. The suitability of the approach could be confirmed by comparing the funnels and folding routes for 5 sequences, where much larger folding times were estimated for sequences known to be difficult to fold. Furthermore, a doubly degenerate sequence yielded a funnel with two native states, as expected.

Since the methods employed are entirely generic, this approach is a potential tool to be used in association with other methods that efficiently probe the energy landscape, such as diffusion-map-directed MD (DM-d-MD) [51], disconnectivity graphs [20] and metadynamics [52]. The method was tested in a simple lattice model, in which the minima were sampled with variable time intervals. It will be straight forward to apply this methodology to realistic models and more meaningful sampling methods, such as those used by Wales [20], [21], [25]. In particular, our method may be helpful to probe details of folding trajectories and effects of mutation in the study of metastable states. As applications, previous work using disconnectivity graphs analyzed the potential energy landscapes of proteins involving gatekeeper residues [33], [53], [54]. By probing the gatekeeper residue contacts using our method we expect to be able to shed light into the nature of these peculiar conformational states.

## Methods

### Model

In this lattice model, a globular protein is modeled as a simplified heteropolymer made up of 27 monomers (or beads) covalently bonded. The monomers are placed on the vertices of a cubic lattice. These models are capable of accounting for several features of protein folding [42], where the most compact (folded) structure is a 3×3×3 cube. One contact is defined for two monomers that are at nearest-neighbor distances but not connected covalently. In the lattice model the maximum number of contacts is 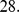 The energy of the system is given by 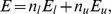 where 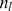 is the number of (non-covalent) contacts of like monomers and 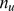 is the number of contacts between distinct monomers. The folding kinetics is performed with the Metropolis algorithm in a Monte Carlo simulation with typical motions in polymers [42]. Here we use a low hydrophobicity regime with 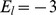 and 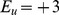 in arbitrary units. This regime was chosen to mimic the folding behavior where the sequence evolves toward its native state without going through a hydrophobic collapse [43], [55]. Five sequences were chosen for the analysis, which exhibit very distinct features, as indicated in Table 1. For each conformation, the free energy was calculated as a function of the parameter 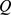 (See Figure S1 in the Supporting Information). The data collected for the projection is restricted to conformations from around the transition state 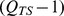 to the native state 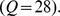 The simulation temperature was set to 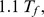 in order for the conformational space to be visited as thoroughly as possible, thus avoiding the sequence having to spend long times in its native state. Local minima were obtained within time intervals segmented along the Monte Carlo trajectories. 4 time intervals were used: 30, 100, 300 and 1000 Monte Carlos steps (MCs). For each interval, the total time was set so that 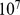 minima were obtained. The conformation at each local minimum was stored in a 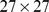 binary matrix representing all the contacts. The conformational matrix is symmetrical and an element 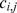 is 1 if there is a contact between monomers 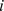 and 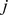 and 0 otherwise.

### Metric

The projection of these multidimensional data was performed using a metric based on the conformational similarity (Jaccard index) and dissimilarity (Jaccard distance), referred to as the structural measurement: 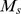 (Eq. 2). We also tested a dynamic measurement in which the number of intermediate minima for going from one conformation to the other was taken into account. This latter metric was named dynamic measurement 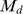 (Eq. 3). Using these measurements one may calculate a normalized effective distance between any two conformations,
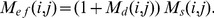
(4)


### Projection

Our goal is not to develop a technique for dimensionality reduction. We want to visualize the similarity between conformations according to our metric. Since the information of structures occurs in a multidimensional space, there is a need for projection into a lower dimension. As with any projection technique, we can create the projection in up to three dimensions [56]. The choice of two dimensions is simply for the ease of data interpretation. 3D projections are very difficult to interpret due to occlusions and overlaps which, in most cases, do not bring real gain compared to 2D [57].

The projection onto a 2D plot was made using the distance matrix with the Force-Scheme method [45], where the objects are initially placed in random positions, and then attraction and repulsion forces between the objects take the system to equilibrium according to a chosen heuristics. Here, the system was initialized with the conformation energies, which proved more efficienct for convergence of the method. After the first placement of the objects, iterations within the Force-Scheme method are performed to preserve similarity in the original space into the projected space. In the first iteration, for each projected point 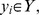 (where 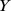 is the input dataset) a vector is calculated 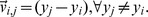 Then 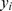 is moved in the 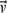 direction by a step 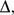 defined as:
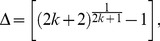
(5)where 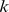 is the number of previous iterations. After an iteration, each object should be moved closer to its similar ones until the system converges. The number of iterations may be defined arbitrarily or the scheme may be stopped when a threshold is reached. Here the process was stopped when the difference in distances for a given object between two consecutive iterations was below a threshold of 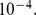 In order to build the 3D funnel, the points in the 2D projection are shifted along a perpendicular axis according to their energies, thus generating a 3D structure where the lowest-energy states are placed on the bottom. We also performed tests with one of the most precise projection techniques in terms of distance preservations, referred to as Classical Multidimensional Scaling (MDS) [56]. The results were similar to those produced by the Force-Scheme in terms of distributing the points on the plane according to the similarity between conformations, with the final shape of the funnels also being very similar. The MDS technique, however, is much more costly in computational time, and in some cases ordinary microcomputers lack the power to obtain the funnels. Therefore, we opted for the Force-Scheme approach, which is much faster and allows one to process thousands of conformations in a few minutes with a simple PC.

## Supporting Information

Figure S1
**Free energy** ***vs*** **Native contacts (Q).** Free energy as a function of native contacts (Q) for four protein-like sequences A, Af, B and C. The simulation was performed at the folding transition temperature 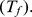

(TIF)

Figure S2 **Mean first-passage times.** Mean first-passage times as a function of the logarithm of the number of local minima needed to reach the native state. Note that the two proteins with high Zscore (A and B sequences), on average, fold more quickly. In contrast, in the sequences with a low Zscore (Af and C sequences), the number of conformations necessary to reach the native state is much greater.
(TIF)

Figure S3 **Visualization in two dimensions for all time intervals for sequence A.** a) 30 MCs; b) MC 100; c) 300 MCs and d) 1000 MC.
(TIF)

Figure S4 **Visualization in two dimensions for all time intervals of sequence Af.** a) 30 MCs; b) MC 100; c) 300 MCs and d) 1000 MC.
(TIF)

Figure S5 **Visualization in two dimensions for all time intervals for sequence B.** a) 30 MCs; b) MC 100; c) 300 MCs and d) 1000 MC.
(TIF)

Figure S6 **Visualization in two dimensions for all time intervals for sequence C.** a) 30 MCs; b) MC 100; c) 300 MCs and d) 1000 MC.
(TIF)

Figure S7 **3D visualization of the funnel for sequence C.** A profile of the funnel is shown on the left, while details of the internal and external parts of the funnel are shown on the right.
(TIF)

Figure S8 **3D visualization of the funnel for sequence D.** A profile of the funnel is shown on the left, while details of the internal and external parts of the funnel are shown on the right.
(TIF)

## References

[1] Anfinsen CB (1973) Principles that govern the folding of protein chains. Science (New York, NY) 181: 223–230. 10.1126/science.181.4096.223 4124164

[2] Levinthal C (1968) Are there pathways for protein folding? Extrait du Journal de Chimie Physique 65.

[3] Bryngelson JD, Onuchic JN, Socci ND, Wolynes PG (1995) Funnels, pathways, and the energy landscape of protein folding: A synthesis. Proteins: Structure, Function, and Bioinformatics 21: 167–195. 10.1002/prot.340210302 7784423

[4] Leopold PE, Montal M, Onuchic JN (1992) Protein folding funnels: a kinetic approach to the sequence-structure relationship. Proceedings of the National Academy of Sciences of the United States of America 89: 8721–8725. 1528885 10.1073/pnas.89.18.8721 PMC49992

[5] Thirumalai D, O'Brien EP, Morrison G, Hyeon C (2010) Theoretical perspectives on protein folding. Annual Review of Biophysics 39: 159–183. 10.1146/annurev-biophys-051309-103835 20192765

[6] Dill KA, Ozkan SB, Shell MS, Weikl TR (2008) The protein folding problem. Annual Review of Biophysics 37: 289–316. 10.1146/annurev.biophys.37.092707.153558 PMC2443096 18573083

[7] Onuchic JN, Luthey-Schulten Z, Wolynes PG (1997) Theory of protein folding: the energy landscape perspective. Annual review of physical chemistry 48: 545–600. 10.1146/annurev.physchem.48.1.545 9348663

[8] Klimov DK, Thirumalai D (1998) Linking rates of folding in lattice models of proteins with underlying thermodynamic characteristics. The Journal of Chemical Physics 109: 4119–4125.

[9] Sabelko J, Ervin J, Gruebele M (1999) Observation of strange kinetics in protein folding. Proceedings of the National Academy of Sciences of the United States of America 96: 6031–6036. 10339536 10.1073/pnas.96.11.6031 PMC26830

[10] Nymeyer H, García AE, Onuchic JN (1998) Folding funnels and frustration in off-lattice minimalist protein landscapes. Proceedings of the National Academy of Sciences 95: 5921–5928. 10.1073/pnas.95.11.5921 PMC34496 9600893

[11] Onuchic JN, Nymeyer H, García AE, Chahine J, Socci ND (2000) The energy landscape theory of protein folding: insights into folding mechanisms and scenarios. Advances in protein chemistry 53: 87–152. 10751944 10.1016/s0065-3233(00)53003-4

[12] Schuler B, Lipman EA, Eaton WA (2002) Probing the free-energy surface for protein folding with single-molecule uorescence spectroscopy. Nature 419: 743–747. 12384704 10.1038/nature01060

[13] Lee CL, Stell G, Wang J (2003) First-passage time distribution and non-markovian diffusion dynamics of protein folding. The Journal of Chemical Physics 118: 959–968.

[14] Chavez LL, Onuchic JN, Clementi C (2004) Quantifying the roughness on the free energy landscape: entropic bottlenecks and protein folding rates. Journal of the American Chemical Society 126: 8426–8432. 15237999 10.1021/ja049510+

[15] Wang J, Oliveira RJ, Chu X, Whitford PC, Chahine J, et al (2012) Topography of funneled landscapes determines the thermodynamics and kinetics of protein folding. Proceedings of the National Academy of Sciences 109: 15763–15768. 10.1073/pnas.1212842109 PMC3465441 23019359

[16] Zhuravlev PI, Papoian GA (2010) Protein functional landscapes, dynamics, allostery: a tortuous path towards a universal theoretical framework. Quarterly Reviews of Biophysics 43: 295–332. 20819242 10.1017/S0033583510000119

[17] Potoyan DA, Papoian GA (2011) Energy landscape analyses of disordered histone tails reveal special organization of their conformational dynamics. Journal of the American Chemical Society 133: 7405–7415. 21517079 10.1021/ja1111964

[18] Itzhaki LS, Otzen DE, Fersht AR (1995) The structure of the transition state for folding of chymotrypsin inhibitor 2 analysed by protein engineering methods: evidence for a nucleationcondensation mechanism for protein folding. Journal of molecular biology 254: 260–288. 7490748 10.1006/jmbi.1995.0616

[19] Clementi C, Nymeyer H, Onuchic JN (2000) Topological and energetic factors: what determines the structural details of the transition state ensemble and “en-route” intermediates for protein folding? an investigation for small globular proteins. Journal of molecular biology 298: 937–953. 10801360 10.1006/jmbi.2000.3693

[20] Wales DJ (2010) Energy landscapes: some new horizons. Current Opinion in Structural Biology 20: 3–10. 20096562 10.1016/j.sbi.2009.12.011

[21] Wales D (2003) Energy Landscapes: Applications to Clusters, Biomolecules and Glasses. Cambridge University Press.

[22] Shan Y, Arkhipov A, Kim ET, Pan AC, Shaw DE (2013) Transitions to catalytically inactive conformations in EGFR kinase. Proceedings of the National Academy of Sciences of the United States of America 110: 7270–7275. 23576739 10.1073/pnas.1220843110 PMC3645566

[23] Dobson CM (2003) Protein folding and misfolding. Nature 426: 884–890. 14685248 10.1038/nature02261

[24] Reddy AS, Wang L, Singh S, Ling YL, Buchanan L, et al (2010) Stable and metastable states of human amylin in solution. Biophysical Journal 99: 2208–2216. 20923655 10.1016/j.bpj.2010.07.014 PMC3042569

[25] Wales DJ (2012) Decoding the energy landscape: extracting structure, dynamics and thermodynamics. Philosophical transactions Series A, Mathematical, physical, and engineering sciences 370: 2877–2899. 10.1098/rsta.2011.0208 22615466

[26] Wales DJ, Bogdan TV (2006) Potential energy and free energy landscapes. The Journal of Physical Chemistry B 110: 20765–20776. 17048885 10.1021/jp0680544

[27] Becker OM, Karplus M (1997) The topology of multidimensional potential energy surfaces: Theory and application to peptide structure and kinetics. The Journal of Chemical Physics 106: 1495–1517.

[28] Becker OM (1997) Quantitative visualization of a macromolecular potential energy “funnel”. Journal of Molecular Structure: THEOCHEM 398–399: 507–516.

[29] Wales DJ, Miller MA, Walsh TR (1998) Archetypal energy landscapes. Nature 394: 758–760.

[30] Miller MA, Doye JPK, Wales DJ (1999) Structural relaxation in atomic clusters: Master equation dynamics. Physical Review E 60: 3701–3718. 10.1103/physreve.60.3701 11970203

[31] Doye J, Miller M, Wales D (1999) The double-funnel energy landscape of the 38-atom lennard-jones cluster. The Journal of Chemical Physics 110: 6896.

[32] Doye J, Miller M, Wales D (1999) Evolution of the potential energy surface with size for lennardjones clusters. The Journal of Chemical Physics 111: 8417.

[33] Wales DJ, Dewsbury PEJ (2004) Effect of salt bridges on the energy landscape of a model protein. The Journal of Chemical Physics 121: 10284–10290. 15549905 10.1063/1.1810471

[34] Miller MA, Wales DJ (1999) Energy landscape of a model protein. The Journal of Chemical Physics 111: 6610–6616.

[35] Evans DA, Wales DJ (2003) The free energy landscape and dynamics of met-enkephalin. The Journal of Chemical Physics 119: 9947–9955.

[36] Krivov SV, Karplus M (2002) Free energy disconnectivity graphs: Application to peptide models. The Journal of Chemical Physics 117: 10894–10903.

[37] Noé F, Fischer S (2008) Transition networks for modeling the kinetics of conformational change in macromolecules. Current Opinion in Structural Biology 18: 154–162. 18378442 10.1016/j.sbi.2008.01.008

[38] Prada-Gracia D, Gómez-Gardeñes J, Echenique P, Falo F (2009) Exploring the free energy landscape: From dynamics to networks and back. PLoS Comput Biol 5: e1000415. 19557191 10.1371/journal.pcbi.1000415 PMC2694367

[39] Noé F, Horenko I, Schutte C, Smith J (2007) Hierarchical analysis of conformational dynamics in biomolecules: Transition networks of metastable states. The Journal of Chemical Physics 126.. 10.1063/1.2714539 17461666

[40] Rao F, Caisch A (2004) The protein folding network. Journal of molecular biology 342: 299–306. 15313625 10.1016/j.jmb.2004.06.063

[41] Dickson A, Brooks CL (2013) Native states of fast-folding proteins are kinetic traps. Journal of the American Chemical Society 135: 4729–4734. 23458553 10.1021/ja311077u PMC3619186

[42] Socci ND, Onuchic JN (1995) Kinetic and thermodynamic analysis of proteinlike heteropolymers: Monte carlo histogram technique. The Journal of Chemical Physics 103: 4732–4744.

[43] Socci ND, Onuchic JN, Wolynes PG (1998) Protein folding mechanisms and the multidimensional folding funnel. Proteins 32: 136–158. 9714155

[44] Garstecki P, Hoang TX, Cieplak M (1999) Energy landscapes, supergraphs, and folding funnel in spin systems. Physical Review E 60: 3219–3226. 10.1103/physreve.60.3219 11970130

[45] Tejada E, Minghim R, Nonato LG (2003) On improved projection techniques to support visual exploration of multi-dimensional data sets. Information Visualization 2: 218–231.

[46] Choi S, Cha S, Tappert C (2010) A survey of binary similarity and distance measures. Journal on Systemics, Cybernetics and Informatics 8: 43–48.

[47] Tan PN, Steinbach M, Kumar V (2005) Introduction to data mining. Boston: Pearson Addison Wesley.

[48] Wales DJ (2006) Energy landscapes: calculating pathways and rates. International Reviews in Physical Chemistry 25: 237–282.

[49] Wales DJ (2002) Discrete path sampling. Molecular Physics 100: 3285–3305.

[50] Wales DJ (2004) Some further applications of discrete path sampling to cluster isomerization. Molecular Physics 102: 891–908.

[51] Zheng W, Rohrdanz MA, Clementi C (2013) Rapid exploration of configuration space with diffusion-map-directed molecular dynamics. The journal of physical chemistry B. 10.1021/jp401911h PMC3808479 23865517

[52] Laio A, Parrinello M (2002) Escaping free-energy minima. Proceedings of the National Academy of Sciences 99: 12562–12566. 10.1073/pnas.202427399 PMC130499 12271136

[53] Otzen DE, Oliveberg M (1999) Salt-induced detour through compact regions of the protein folding landscape. Proceedings of the National Academy of Sciences 96: 11746–11751. 10.1073/pnas.96.21.11746 PMC18357 10518521

[54] Kurnik M, Hedberg L, Danielsson J, Oliveberg M (2012) Folding without charges. Proceedings of the National Academy of Sciences 109: 5705–5710. 10.1073/pnas.1118640109 PMC3326513 22454493

[55] Chahine J, Nymeyer H, Leite VBP, Socci ND, Onuchic JN (2002) Specific and nonspecific collapse in protein folding funnels. Physical review letters 88: 168101. 11955268 10.1103/PhysRevLett.88.168101

[56] Cox TF, Cox MAA (2010) Multidimensional Scaling, Second Edition. CRC Press.

[57] Ware C (2001) Designing with a 2 1/2d attitude. Information Design Journal 10: 2001.

[58] Dima RI, Banavar JR, Cieplak M, Maritan A (1999) Statistical mechanics of protein-like heteropolymers. Proceedings of the National Academy of Sciences 96: 4904–4907. 10.1073/pnas.96.9.4904 PMC21789 10220391

[59] Abkevich VI, Gutin AM, Shakhnovich EI (1994) Free energy landscape for protein folding kinetics: Intermediates, traps, and multiple pathways in theory and lattice model simulations. The Journal of Chemical Physics 101: 6052–6062.

