# Supplementary figures and images for: Visualization of Protein Folding Funnels in Lattice Models
Source: PLoS One. 2014 Jul 10;9(7):e100861. doi: 10.1371/journal.pone.0100861 (PMC4091862; doi:10.1371/journal.pone.0100861)

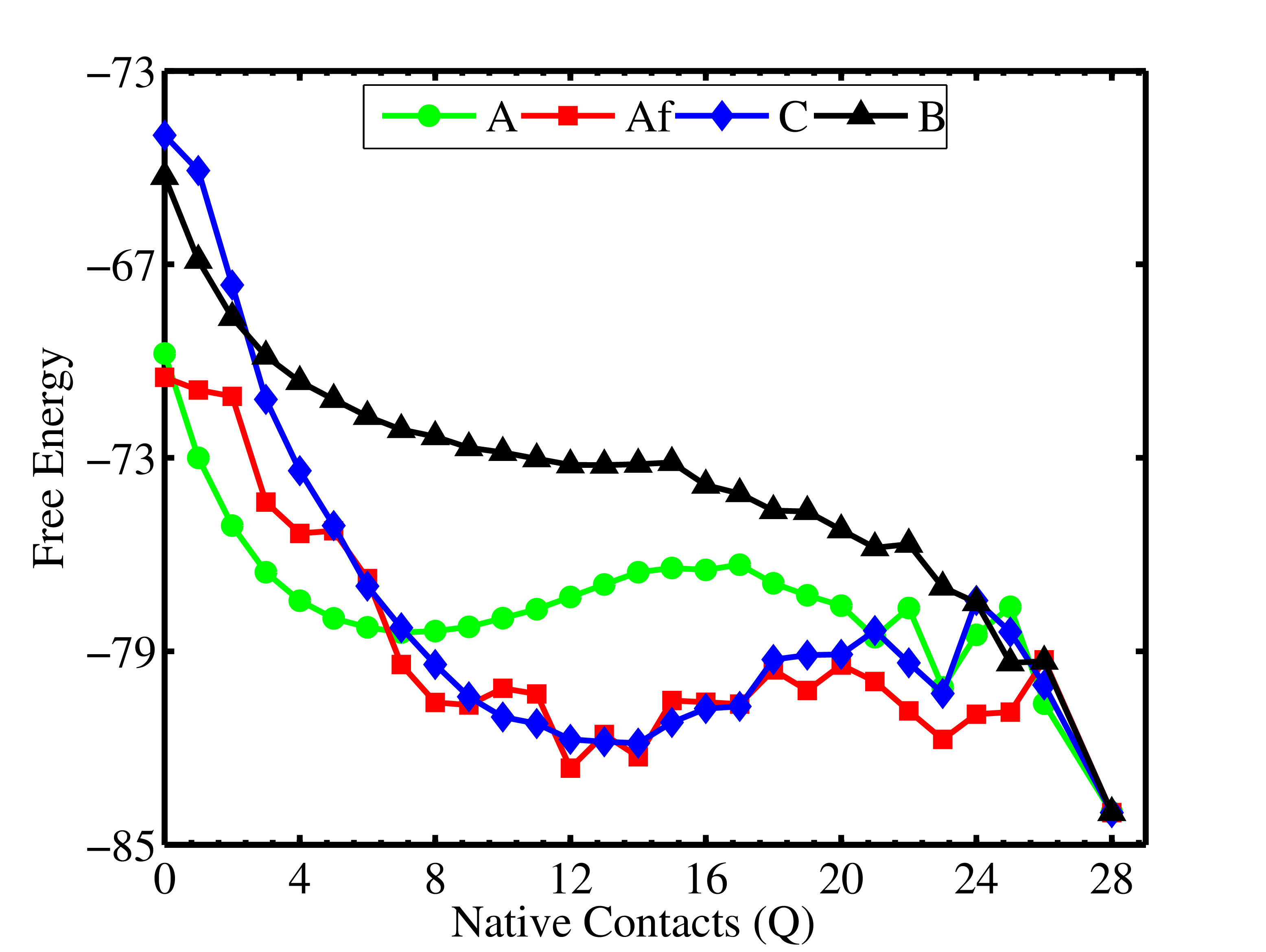

Supplement: Figure S1 — Free energy vs Native contacts (Q). Free energy as a function of native contacts (Q) for four protein-like sequences A, Af, B and C. The simulation was performed at the folding transition temperature (TIF) [file pone.0100861.s001.tif]

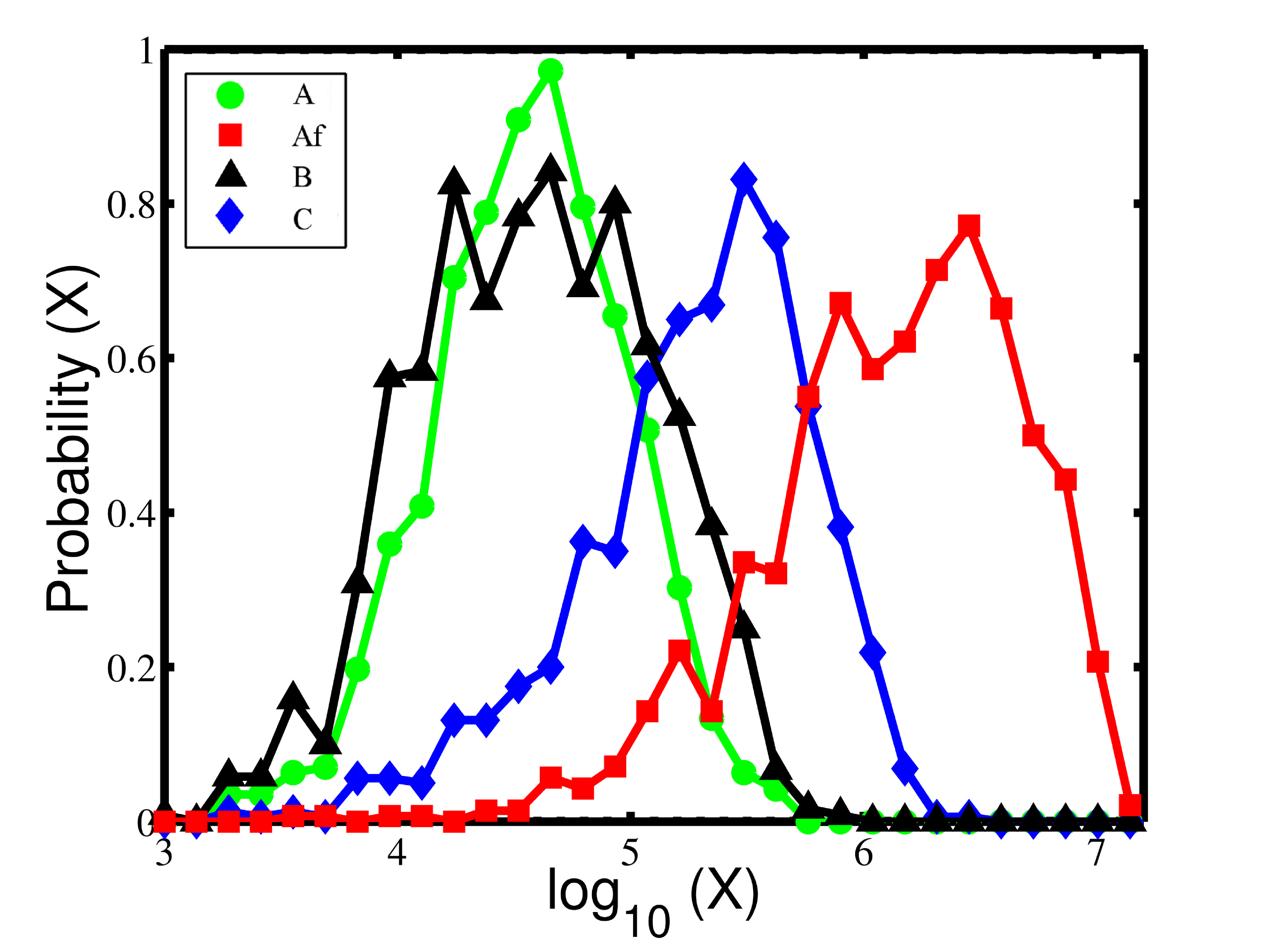

Supplement: Figure S2 — Mean first-passage times. Mean first-passage times as a function of the logarithm of the number of local minima needed to reach the native state. Note that the two proteins with high Zscore (A and B sequences), on average, fold more quickly. In contrast, in the sequences with a low Zscore (Af and C sequences), the number of conformations necessary to reach the native state is much greater. (TIF) [file pone.0100861.s002.tif]

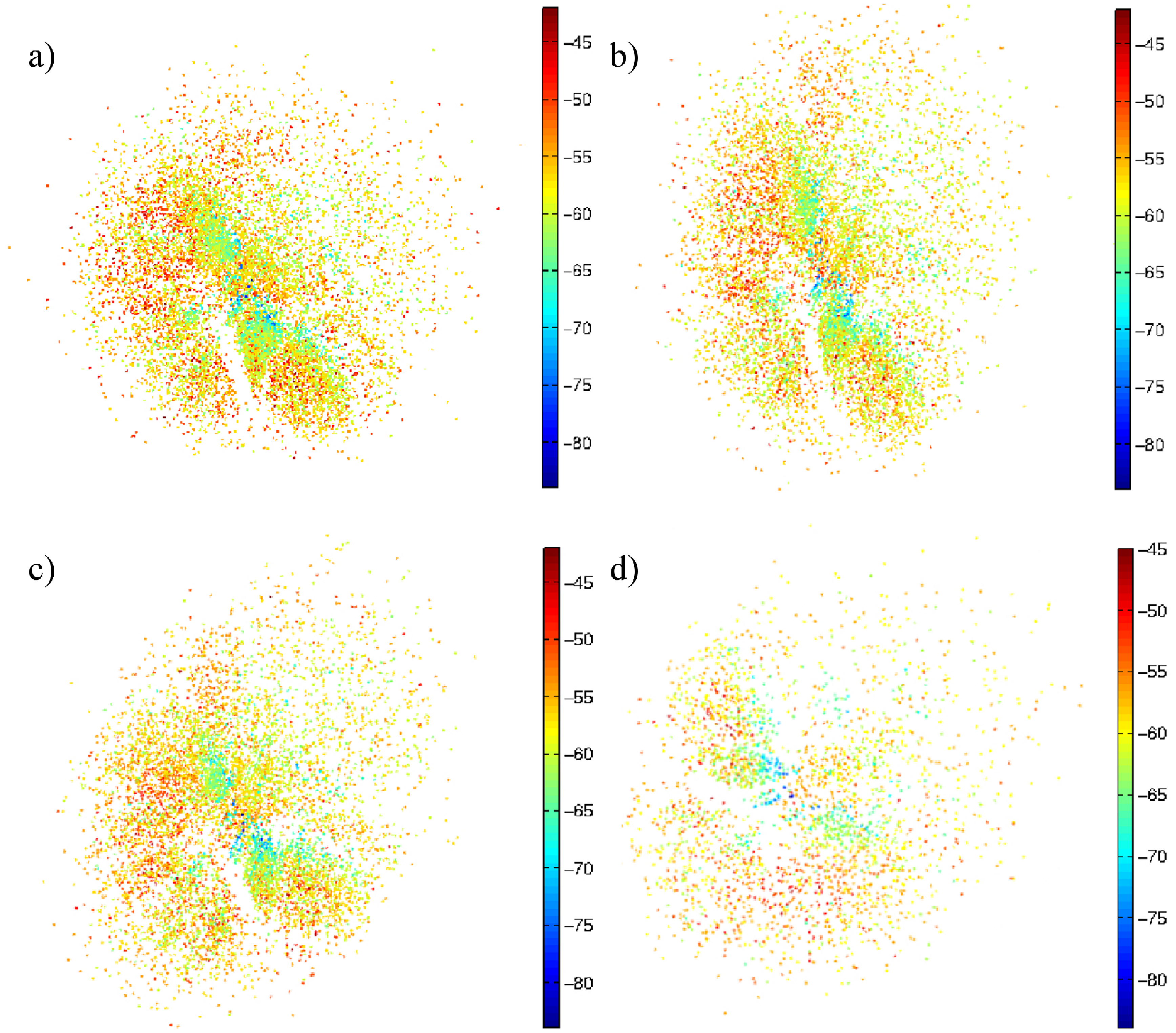

Supplement: Figure S3 — Visualization in two dimensions for all time intervals for sequence A. a) 30 MCs; b) MC 100; c) 300 MCs and d) 1000 MC. (TIF) [file pone.0100861.s003.tif]

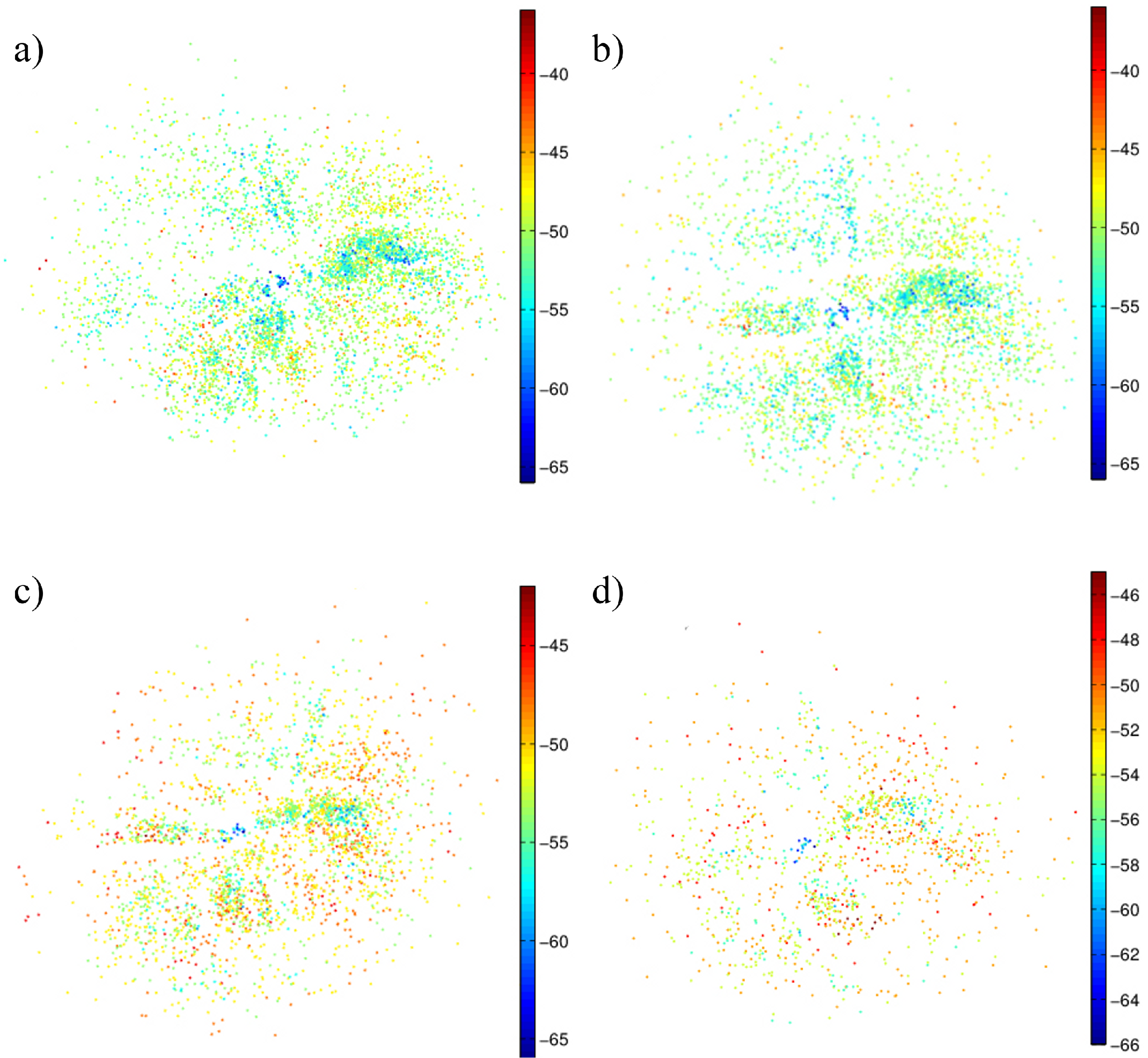

Supplement: Figure S4 — Visualization in two dimensions for all time intervals of sequence Af. a) 30 MCs; b) MC 100; c) 300 MCs and d) 1000 MC. (TIF) [file pone.0100861.s004.tif]

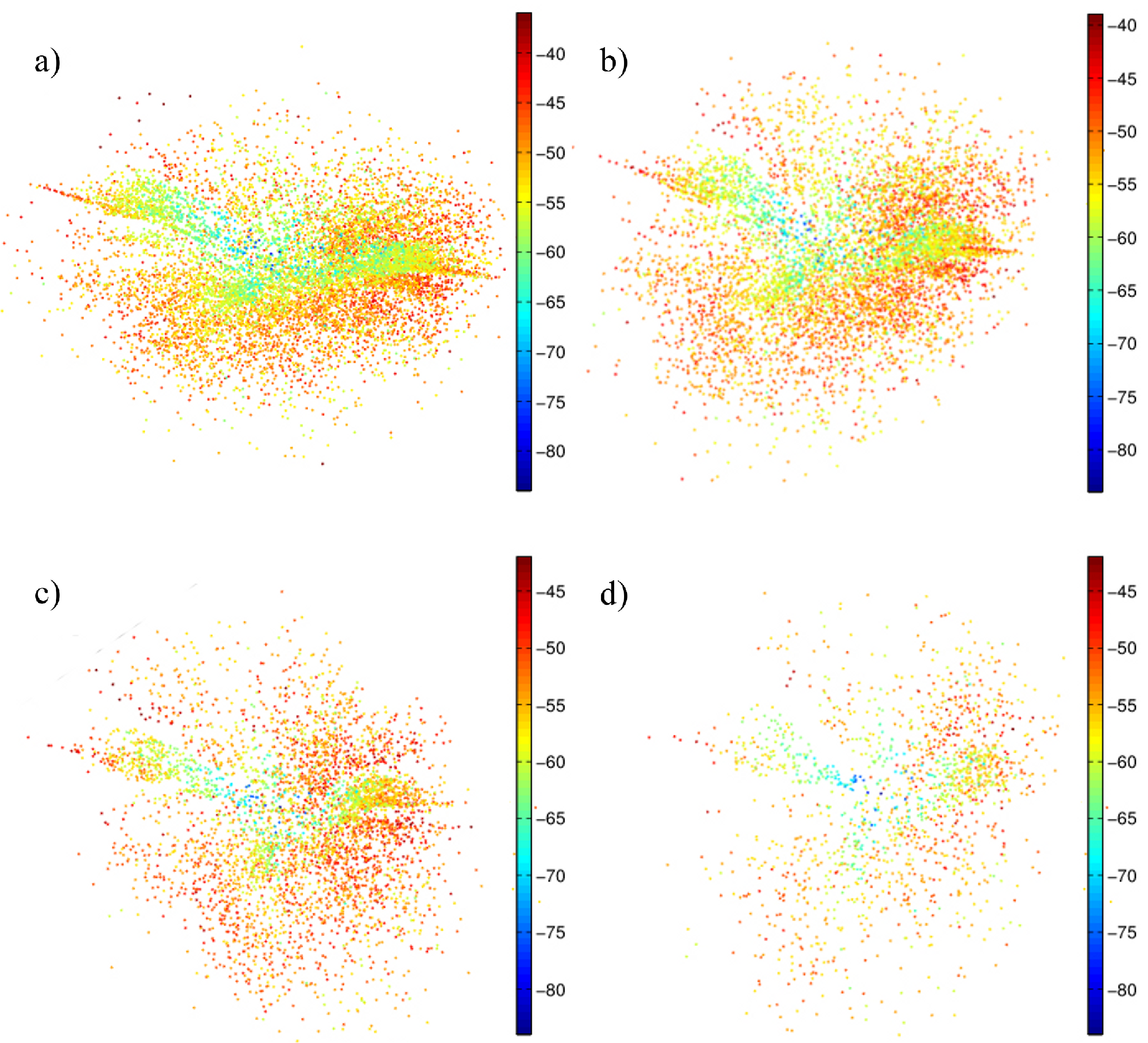

Supplement: Figure S5 — Visualization in two dimensions for all time intervals for sequence B. a) 30 MCs; b) MC 100; c) 300 MCs and d) 1000 MC. (TIF) [file pone.0100861.s005.tif]

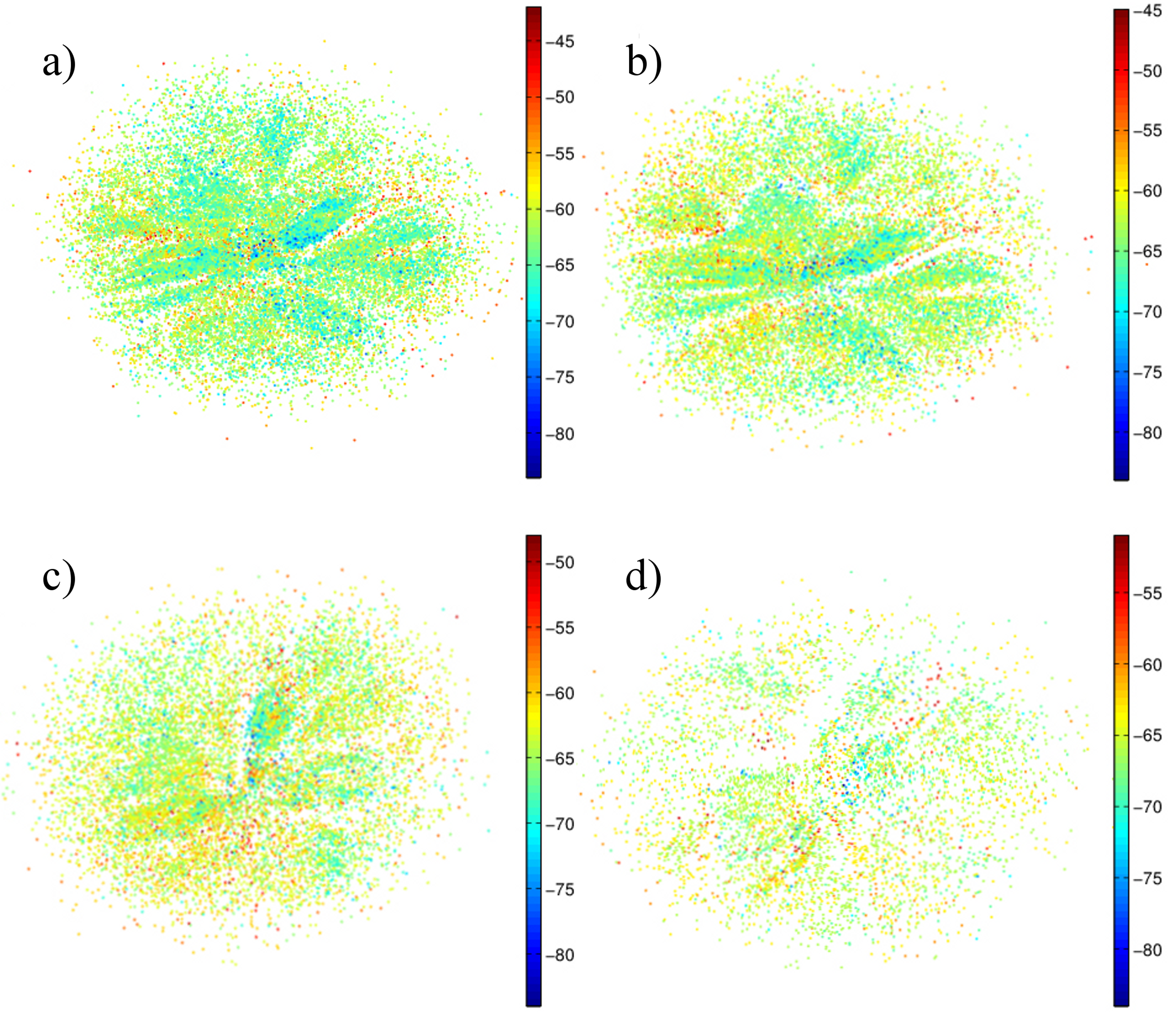

Supplement: Figure S6 — Visualization in two dimensions for all time intervals for sequence C. a) 30 MCs; b) MC 100; c) 300 MCs and d) 1000 MC. (TIF) [file pone.0100861.s006.tif]

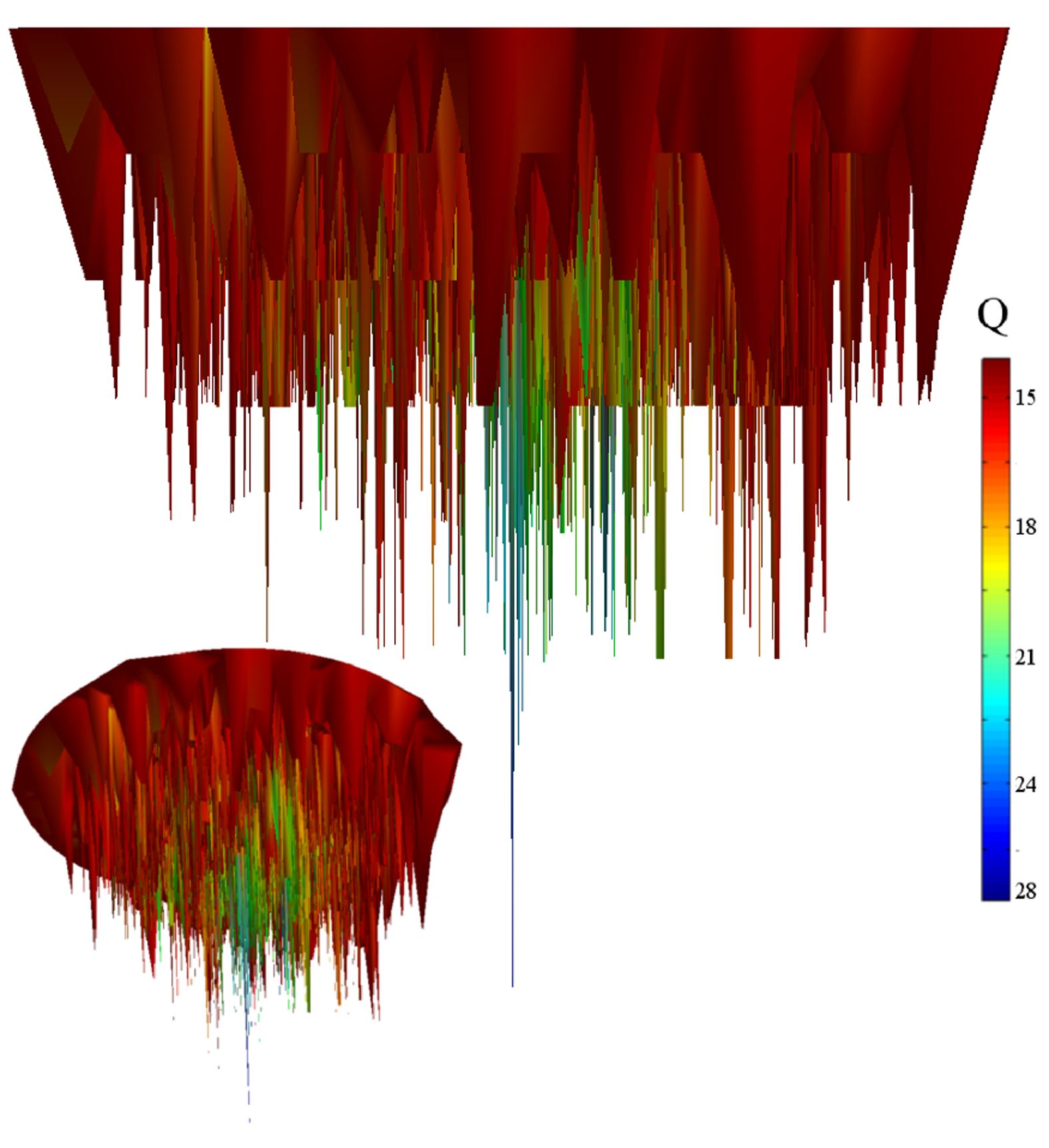

Supplement: Figure S7 — 3D visualization of the funnel for sequence C. A profile of the funnel is shown on the left, while details of the internal and external parts of the funnel are shown on the right. (TIF) [file pone.0100861.s007.tif]

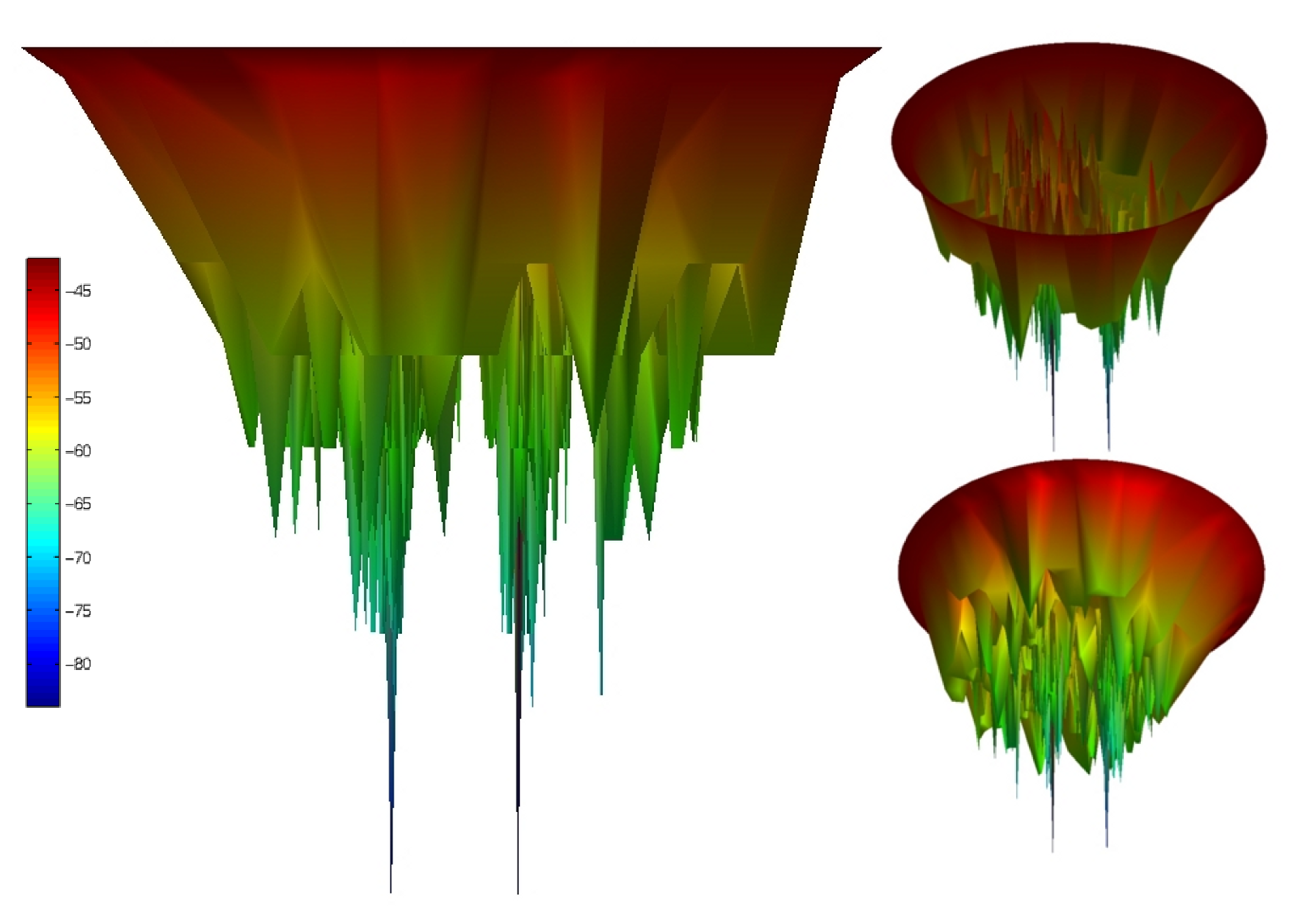

Supplement: Figure S8 — 3D visualization of the funnel for sequence D. A profile of the funnel is shown on the left, while details of the internal and external parts of the funnel are shown on the right. (TIF) [file pone.0100861.s008.tif]
